# Mental health implications of gender affirming care: bridging the communication and supportive gap

**DOI:** 10.1097/MS9.0000000000004473

**Published:** 2025-12-11

**Authors:** Sidhant Ochani, Syeda Ilsa Aaqil, Alishba Adnan, Abubakar Nazir, Arpit Mago, Sumran Azam, Kaleem Ullah, Md. Asaduzzaman Nur

**Affiliations:** a Department of Medicine, Khairpur Medical College, Khairpur Mir’s, Pakistan; b Department of Medicine, Jinnah Sindh Medical University, Karachi, Pakistan; c Department of Medicine, Karachi Medical and Dental College, Karachi, Pakistan; d Department of Medicine, King Edward Medical University, Lahore, Pakistan; e Department of Medicine, Jawarharlal Nehru Medical College, Belgaum, Karnataka, India; f Department of Hepatobiliary Surgery, Al-Amiri Hospital, Kuwait; g Faculty of HPB Surgery, Enam Medical College, Dhaka, Bangladesh

**Keywords:** gender affirming care, gender dysphoria, gender minority, LGBTQIA, mental health

## Abstract

Gender-affirming healthcare is a comprehensive approach that aims to support individuals in transitioning from the gender assigned to them at birth to their affirmed gender, aligning with their gender identity. This type of care involves medically necessary and scientifically supported interventions within a multidisciplinary framework. Gender dysphoria, also known as gender incongruence or transsexuality, refers to the experience of a mismatch between an individual’s biological sex and their internal sense of gender, known as gender identity. Gender identity can encompass a range of expressions and may include identifying as neither exclusively male nor female, allowing for a spectrum of gender diversity. Studies conducted in the past have revealed alarming statistics, showing that transgender and gender-diverse (TGD) individuals face a 41% lifetime prevalence of suicide attempts, a lifetime prevalence of binge drinking ranging from 7% to 61%, and a 33% prevalence of tobacco use. The increased vulnerability to adverse mental health outcomes among TGD individuals can be attributed to a variety of factors, including the presence of stigma, discrimination, pathologization, economic marginalization, violence, and the distress arising from a misalignment between their gender identity and societal expectations rooted in their assigned sex at birth. Research reveals that a significant proportion of gender and sexual minority adults have experienced discrimination from healthcare providers, with approximately 8% of sexual minorities and 25% of transgender individuals reporting being denied healthcare services. This review outlines the bridging of the communication and supportive gap in gender-affirming care to improve patient outcomes and overall quality of care.

## Introduction

Gender-affirming healthcare is a multidisciplinary approach that supports individuals transitioning from their sex assigned at birth to their affirmed gender, aligning with their gender identity[1]. Gender dysphoria, also called gender incongruence, is the distress caused by a mismatch between biological sex and internal gender identity[2]. A transgender woman identifies as female but was assigned male at birth, while a transgender man identifies as male but was assigned female. “Sex” refers to biological traits such as anatomy and genetics, whereas “gender” reflects internal identity and may include nonbinary expressions beyond the male-female binary[2]. Key terms used for gender minority patients have been discussed in Table 1.

**HIGHLIGHTS**

- Telehealth has improved access to gender-affirming care during the COVID-19 pandemic by reducing costs, increasing flexibility, and reaching underserved populations, especially youth without parental support.
- Clear and respectful communication, including the correct use of pronouns and gender-neutral language, fosters trust, improves treatment adherence, and enhances health outcomes for TGNC individuals.
- Systemic changes, such as expanding insurance coverage, reducing discrimination, and increasing provider education, are crucial to addressing financial, structural, and social barriers faced by TGNC patients.

**Table 1:** Key terms used for gender minority patients

Transgender	Individuals are those whose gender identity does not align with the sex they were assigned at birth. For example, a person assigned female at birth who identifies as male is a transgender man, and vice versa.
Nonbinary	Individuals identify outside the traditional categories of male and female. They may have a gender identity that is a combination of both genders, or they may reject the concept of gender altogether.
Genderqueer	Umbrella term used by individuals who reject or challenge traditional binary gender identities. It can encompass various nonbinary identities and expressions.
Affirmed gender	Affirmed gender refers to a person’s true gender identity, which may be different from their assigned sex at birth. When individuals undergo a social and/or medical transition to align with their affirmed gender, it is known as gender affirmation or transition.
Gender fluid	Defined as a gender identity that is flexible or changes over time or in response to circumstances. Changes in gender identity or gender expression can also take place. A person who identifies as gender fluid may express themselves in a variety of ways throughout their lifespan or in combination with other gender markers.

During puberty, transgender individuals often experience significant psychological distress due to this misalignment and may seek gender-affirming interventions to alleviate it[3]. These interventions aim to align the affirmed gender with the authentic self. Transgender and gender-diverse (TGD) individuals face disproportionately high mental health burdens, including a 41% lifetime prevalence of suicide attempts[4], binge drinking rates up to 61%, and a 33% prevalence of tobacco use[5]. These outcomes stem from stigma, discrimination, marginalization, violence, and distress from incongruence between identity and societal expectations[5].

Transphobia further worsens disparities in healthcare access and outcomes. Many providers lack training in transgender care, and bias undermines competence even with additional education[6]. Discrimination is common, with about 8% of sexual minorities and 25% of transgender individuals denied healthcare[6]. This review explores ways to bridge communication and supportive gaps in gender-affirming care to improve outcomes and quality of care. This study has been reported in line with the TITAN guidelines 2025[7].

## Gender affirmation: understanding the spectrum of care

Gender-affirming care addresses the social, mental, and medical well-being of TGD individuals through a tailored, holistic approach across disciplines, including endocrinology, surgery, primary care, reproductive and sexual health, and mental health[8]. Interventions may include puberty suppression, hormone therapy, and gender-affirming surgeries, though not all individuals require every component. Effective communication and coordinated care are essential, particularly in resource-limited settings where provider networks can help bridge service gaps[8].

Hormone therapy, using estrogen and antiandrogens for transfeminine individuals and testosterone for transmasculine individuals, is a core element of medical gender affirmation and significantly influences physical appearance^[9,10]^. Evidence shows that hormone therapy improves quality of life and reduces depression and anxiety across genders and age-groups[11].

Gender-affirming surgery further enhances mental health outcomes, significantly reducing severe psychological distress, suicidal ideation, and smoking rates among recipients[5]. These findings highlight the importance of ensuring access to surgical interventions and implementing policies that expand and protect such care for TGD communities[5].

## Considerations for personalized treatment plans

LGBTI individuals face substantial disadvantages within healthcare systems that impede patient-centered care. Despite recommendations to consider psychological, physical, social, and cultural factors during diagnosis and treatment, this is rarely practiced. Strict eligibility criteria imposed by clinicians, a practice known as “gatekeeping,” create significant barriers, both physical and mental, for transgender patients[12]. This reinforces misconceptions that seeking gender-affirming care is a mental disorder[13].

The World Professional Association for Transgender Health Standards of Care version 8 advocates shifting from the gatekeeping model to an informed consent model, emphasizing the interconnection of medical and mental health needs[12]. Transition-related care, including mental health services, endocrine therapy, and surgeries, is most effective when supported by informed consent and peer support[14]. Individualized care tailored to patient needs, combined with enhanced provider training, is crucial to improving outcomes.

## Mental health implications of gender-affirming care

Transgender individuals often experience high rates of depression, suicidal ideation, and body image dysphoria[15]. These outcomes are closely linked to “minority stress,” the chronic stress arising from stigma, discrimination, and lack of affirmation of one’s gender identity^[16–18]^. Access to a full spectrum of gender-affirming resources, from hormone therapy and surgeries to social changes like hairstyles and clothing, plays a pivotal role in validating identity, strengthening self-concept, and improving psychological well-being[19].

Gender-affirming care for transgender and nonbinary youth is consistently associated with improved mental health. Puberty blockers, hormone therapy, and surgical interventions independently correlate with reduced anxiety, depression, and other adverse outcomes^[20,21]^. Adolescents with access to puberty blockers show lower lifetime prevalence of suicidal thoughts as adults[22]. Tordoff *et al* reported that during the first year of comprehensive gender care, moderate-to-severe depressive symptoms were reduced by 60%, and self-harm or suicidal ideation decreased by 73%[23].

Despite this evidence, legislative restrictions on gender-affirming care are increasing[24]. These laws risk reversing progress in mental health outcomes by denying youth access to essential treatments. Ensuring legal protection and equitable access to gender-affirming interventions is therefore vital to safeguarding mental health in TGD populations.

## Communication and supportive gap in gender-affirming care

Many healthcare providers are unaware that “sexual and gender minorities” (SGM) is the current National Institutes of Health (NIH)-endorsed term, replacing LGBT. This lack of knowledge, along with the misuse of terminology, contributes to a scarcity of research data and inadequate care. Studies reveal methodological gaps, such as unrepresentative sampling, that hinder the understanding of gender minority needs[25].

Stigmatization often begins at home and can escalate to domestic abuse, leaving individuals without financial or emotional support and delaying care-seeking[26]. Policy-level barriers also exist; many hospital admission forms require binary gender selection, and insurance companies often deny coverage when gender markers do not align with assigned sex at birth[26]. Clinician-related factors, including age, political or religious beliefs, limited knowledge, and stigma, further restrict access. Patients frequently bear the burden of educating providers, reducing the time available for addressing medical needs[26].

Gender minorities are disproportionately exposed to violence. In a study of 3798 participants, 23% identified as gender minorities, and 56% reported lifetime violence, with 25% experiencing physical violence and 19% sexual violence in the past year[27]. Healthcare environments also remain unprepared: more than half (54.3%) of providers reported no workplace guidelines for name and pronoun use, 51.2% lacked gender-neutral toilets, and 45.1% had no system to avoid calling out legal names in waiting areas[28].

## Bridging the communication and supportive gap

Improving provider knowledge about the challenges faced by SGM populations, including high rates of depression, anxiety, and eating disorders, is essential[29]. Clinicians should screen for violence in relationships, provide appropriate referrals, and tailor sexual and reproductive healthcare to each patient[29]. Effective counseling builds trust and confidence among SGM patients.

Telehealth offers additional solutions by allowing patients to access care discreetly, avoid unnecessary disclosures, and reduce financial burdens[30]. Despite legal protections, discrimination and victimization remain prevalent, perpetuating health risks and marginalization[31]. Developing comprehensive educational content and structured referral protocols can improve care coordination[32]. Government-issued directories of affirming providers and frequent conferences on gender-affirming topics would enhance provider competence and public awareness[32].

Community-building initiatives and state funding for SGM organizations can increase safety, promote access, and reduce violence[32]. Addressing prejudice requires visible advocacy from subject matter experts and political leaders[32]. Public messaging should highlight the strengths and contributions of SGM individuals rather than perpetuating stigma. Finally, information dissemination must extend beyond crisis-focused messaging to include holistic health promotion[32].

## Intersectionality of gender and other marginalized identities

Providing gender-affirming care requires recognizing the intersection of gender with other marginalized identities such as race, ethnicity, disability, and socioeconomic status[33]. Individuals possess multiple overlapping identities that shape their experiences, influencing health outcomes and access to care[34]. Healthcare providers must understand how cultural and social norms affect patient behavior and how their own biases and stereotypes can influence clinical decisions[34].

Addressing these biases involves creating inclusive environments that promote equitable communication, appropriate referrals, and social inclusion. Dismantling systemic barriers enhances outcomes for individuals from diverse backgrounds[34]. Education and training for families are also crucial to improving the participation of individuals with disabilities and fostering acceptance[34]. Considering intersectionality allows for more holistic, responsive, and equitable care.

## Community support and advocacy

Access to gender-affirming care is critical to the mental well-being of transgender youth. Those denied such care experience significantly higher rates of depression, anxiety, and suicidality than peers with supportive families and clinicians[35]. Early interventions and social transition, aligning gender identity with lived experience, lead to improved mental health, with socially transitioned transgender children showing mental health levels comparable to their cisgender peers[36].

However, restrictive policies remain a major concern. For instance, Arkansas became the first state to ban gender-affirming care for minors, including hormone therapy and surgeries[35]. Such legislation has been widely criticized by human rights organizations for undermining essential healthcare access[35].

Policy interventions can help reduce barriers. Professional organizations should promote culturally competent and affirming care through guidelines, such as those by the American Psychological Association (2015), and publicly oppose discriminatory laws[37]. Employment discrimination must be addressed, as financial insecurity limits healthcare access[37]. Policies at both federal and state levels prohibiting discrimination based on gender identity and expression are essential[37].

Insurance exclusions also create major barriers. Comprehensive reforms should ensure all transgender and gender-nonconforming (TGNC) individuals have health insurance covering gender-affirming care[37]. Eliminating these exclusions and improving healthcare options would significantly improve access and outcomes[37].

Community engagement is another cornerstone of improved care. Building partnerships between healthcare providers and community organizations fosters trust, facilitates outreach, and informs policy. Supporting grassroots advocacy efforts and peer networks can empower transgender individuals and strengthen healthcare delivery systems[32].

By addressing discriminatory policies, improving cultural competence among providers, eliminating insurance barriers, and strengthening community support, healthcare systems can advance equity and promote better outcomes for TGD individuals.

## Accessible and affordable gender-affirming care

Despite progress in policy, TGD individuals remain more likely to be uninsured (14–19%) compared to the general population (11–17%)[38]. As of 2021, 30 states offered explicit coverage for transgender people, but only 18 covered gender-affirming surgeries, and 13 continued to deny such coverage[39]. Insurance disparities extend across specific procedures.

Facial feminization surgery, though effective for alleviating gender dysphoria, is typically classified as cosmetic and not covered^[40,41]^. Chondrolaryngoplasty, which reduces the Adam’s apple, is the most commonly covered procedure (~60% of states)[40]. Hair restoration and removal, important for gender affirmation, are also often excluded[42].

Coverage for chest surgeries is inconsistent: while 98% of insurers cover chest masculinization, only 29% cover chest feminization, often misclassifying it as cosmetic[43]. Genital surgeries like clitoroplasty, labiaplasty, and vaginoplasty are usually covered (~90%), but valvuloplasty is frequently denied as cosmetic[44]. Hormone therapy, a cornerstone of gender-affirming care, is often restricted, with most insurers requiring at least 12 months of therapy before certain surgeries[45].

Insurance limitations also affect imaging services, such as mammography for transmasculine patients, due to mismatches between gender identity and medical coding[46]. Mental health services are critical yet often subject to stringent referral requirements, creating barriers that may drive individuals toward unsafe self-administered hormone use or self-surgery[45]. Fertility preservation, important for those seeking biological parenthood, is rarely covered despite its necessity[47]. Expanded insurance policies that address these gaps are essential to ensure equitable care.

## The COVID-19 pandemic and telehealth

The COVID-19 pandemic intensified existing barriers to gender-affirming care. Lockdowns forced the cancellation or postponement of surgeries deemed “elective” and compelled many transgender individuals to live with unsupportive families, exacerbating gender dysphoria and mental health challenges[19].

Telehealth, the use of digital communication to deliver remote healthcare, emerged as a vital solution[48]. It reduces costs, eliminates travel barriers for rural and low-income patients, and improves flexibility for caregivers. Telemedicine also enhances access for youth lacking parental support; a 2020 survey of 204 individuals aged 12–26 found that nearly half preferred telemedicine for gender-affirming care, especially those without family acceptance[49].

These services offer improved communication, easier access to clinical care, and expanded training opportunities for providers[50]. They also bridge gaps caused by geographic, economic, or legislative barriers, making care more accessible. Continued research, documentation, and quality improvement are needed to optimize telehealth delivery for TGD populations[50].

Telehealth empowers patients to engage with providers on their own terms and has become a cornerstone of care continuity during crises. By integrating telemedicine into long-term healthcare strategies, systems can mitigate disparities and improve outcomes for transgender individuals.

## Importance of clear communication

Clear, respectful communication between healthcare providers and TGD patients is essential for effective, affirming care[51]. Basic practices such as asking about gender identity and pronouns build trust, foster safe clinical spaces, and enable tailored treatment plans^[51,52]^. A lack of formal training in gender diversity among providers underscores the importance of ongoing education to improve competence and patient outcomes[52].

Poor communication often leads to misunderstanding, mistrust, and nonadherence to treatment plans, particularly in chronic disease management[53]. Providers must adopt strategies such as using gender-neutral language, avoiding assumptions, and employing transitional phrasing to clarify the medical relevance of questions[53]. Knowing when and how to apologize for mistakes reinforces compassionate care[53].

Training is needed not only for clinicians but also for support staff, including front desk, nursing, and laboratory personnel, to ensure a welcoming environment[53]. Despite this, many providers fail to ask about patients’ preferred anatomical language, highlighting persistent gaps in practice[53].

Effective communication enhances information flow, improves interventions, boosts safety and morale, and increases patient satisfaction[54]. Cross-cultural care principles, including respect, curiosity, and empathy, further strengthen patient–provider relationships. Providers should document affirmed pronouns accurately in medical records and display visible signs of inclusion, such as affirming signage and inclusive paperwork[51]. Awareness of external resources and referral options is also vital for comprehensive support[51].

In sum, clear and inclusive communication promotes trust, adherence, and better health outcomes for TGD patients, forming the foundation of patient-centered, affirming care.

## Strategies for healthcare providers

Healthcare providers play a crucial role in reducing barriers faced by TGD individuals. They must cultivate cultural competence, address personal biases, and use respectful, affirming language. Terms like “biological” or “real” should be replaced with “assigned male/female at birth,” and distressing terminology should be avoided[51]. Providers should also be flexible with patient-preferred language and explain the relevance of sensitive questions^[51,55,56]^.

Understanding the interpersonal challenges TGD patients face allows providers to create affirming care environments and reduce emotional distress[51]. Education is key: integrating discussions on systemic barriers into clinical training encourages mental health professionals to adopt gender-affirming care principles[57]. Structured training, including case discussions, expert consultations, and clinical experiences, improves provider skills and cultural responsiveness[58].

Healthcare systems can foster inclusivity by implementing gender-inclusive intake forms, updating electronic health records to collect gender identity data, and routinely asking all patients about their gender identity[59]. Expanding mental health services requires culturally responsive primary care, greater availability of gender-affirming professionals, and clear consent processes for information sharing[51].

Creating visibly affirming environments, with safe-space signage, pronoun pins, and inclusive language, helps patients feel welcome. Mandatory all-staff training, written policies promoting gender inclusivity, and proper use of affirmed names and pronouns in public spaces further support inclusivity[51]. Leadership should prioritize hiring TGD providers at all levels, including decision-making positions, to drive systemic change[58].

By combining education, inclusive practices, and institutional commitment, healthcare providers can significantly reduce disparities and improve the quality of care for TGD populations.

## Identifying challenges, barriers, and areas for improvement

TGNC individuals face significant systemic barriers to gender-affirming care. Compared to the general U.S. population, they are more likely to be uninsured (14% vs. 11%), unemployed (15% vs. 5%), and living in poverty (29% vs. 12%)[60]. Nearly one-third report living in poverty, and 42% cannot afford necessary care. Insurance denials and lack of coverage for gender-affirming procedures are common[60].

Within healthcare settings, TGNC individuals frequently encounter discrimination, including misgendering, invasive questioning, and outright denial of care^[61,62]^. Lack of provider knowledge further exacerbates these challenges, especially for nonbinary and genderqueer individuals whose identities are often misunderstood or erased[63]. Financial barriers, lack of primary care access, and restrictive insurance policies also limit care[64]. Other obstacles include fear of disclosure, geographic constraints, age-related consent requirements, long waiting lists, and family resistance, often linked to fertility concerns or reluctance to support gender identity^[37,65]^.

These barriers highlight the urgent need for systemic reforms to ensure equitable, accessible, and comprehensive gender-affirming healthcare for all TGNC individuals.

## Addressing disparities in care for marginalized communities

Lack of provider education and understanding often forces TGNC patients to educate their clinicians[61]. Awareness of nonbinary identities remains especially limited[66]. Bias and stigma, including transphobia and cisnormativity, manifest through denial of care, derogatory remarks, harassment, and even abuse from providers and support staff alike[37]. Such behaviors create power imbalances known as “gatekeeping,” leaving patients disempowered and without resources[37].

Discrimination leads many TGNC individuals to avoid care altogether or seek unsafe alternatives such as unregulated medications[67]. Tackling these disparities requires systemic action: providers must seek ongoing training in gender-affirming care, and institutions should confront stigma through inclusive policies, sensitivity training, and accountability mechanisms[37]. Expanding insurance coverage, reducing financial barriers, and increasing the availability of gender-affirming mental health professionals are crucial steps[37].

Community engagement should include TGNC individuals in policy development, foster support networks, and build partnerships between healthcare systems and community organizations[37]. Empowering communities and creating inclusive healthcare environments are essential for eliminating disparities and improving health outcomes.

## Recommendations

Access to comprehensive mental health support should be ensured by providing services from mental health professionals who have expertise in transgender issues and gender identity. Also, these healthcare professionals must be trained in affirmative approaches to mental healthcare[6]. In order to minimize the barriers to care and promote empowerment, an informed consent model for gender-affirming care should be implemented. It aids in emphasizing the shared decision-making between transgender individuals and healthcare providers[6]. Multidisciplinary care teams should be established that comprise healthcare providers, including endocrinologists, psychologists, social workers, and primary care physicians, for a collaborative approach to warrant holistic care[23]. Conducting regular mental health screenings in the concerned population should be a vital part of the gender-affirming care process. This strategy plays a pivotal role in identifying any already existing mental health issues and warrants adequate support and interventions to treat the condition^[23,68]^. Healthcare providers should be trained to deliver culturally competent care that appreciates and respects the unique experiences and challenges faced by transgender individuals to foster trust and a pragmatic therapeutic relationship[69]. Peer support programs should be developed and promoted that can help in dispensing validation among transgender individuals and also improve their mental health. For reducing mental health discrepancies, educational programs should be initiated, and policies should be implemented to highlight stigma, discrimination, and bias toward transgender individuals. These strategies will help in creating a more inclusive society[69]. Language plays an important role when communicating with transgender individuals. Therefore, inclusive language should be used while interacting with transgender individuals. Assumptions about gender identity should be avoided^70^. Identifying and highlighting the intersectionality of gender identity with various associated aspects of identity, like race, ethnicity, socioeconomic status, and immigration status, can help in addressing the discrimination that may impact mental health outcomes and help in tailoring care accordingly[70]. Transgender individuals often experience significant levels of trauma, including violence, discrimination, and rejection. So, trauma-informed care principles should be integrated to create a safe and supportive environment that acknowledges and addresses trauma[68]. Family, school, and workplace social support are crucial in creating supportive environments for transgender individuals. Providing education and support to loved ones can foster acceptance, decrease familial rejection, and enhance the mental well-being of transgender individuals^[6,23,68]^. Art and expressive therapies can help individuals discover and express their gender identity, cope with challenges, and promote self-discovery and empowerment. For increasing the accessibility and reach of mental health services for transgender individuals residing in underserved areas, digital interventions and telehealth should be leveraged[69]. Longitudinal studies and research on mental health outcomes, comparative effectiveness research, and the development of tailored interventions should be conducted to understand the mental health implications^[70,71]^.

## Data Availability

All data and materials can be requested by the corresponding author, SO, and can be provided as per request.
